# PaR1 secreted by the type IX secretion system is a protective antigen of *Riemerella anatipestifer*

**DOI:** 10.3389/fmicb.2022.1082712

**Published:** 2023-01-11

**Authors:** Jialing Wang, Yan Chen, Xiaohua He, Xiaoli Du, Yongheng Gao, Xinggen Shan, Zhiqun Hu, Qinghai Hu

**Affiliations:** Shanghai Veterinary Research Institute, Chinese Academy of Agricultural Sciences, Shanghai, China

**Keywords:** *Riemerella anatipestifer*, protective antigen, subcellular localization, the type IX secretion system (T9SS), PaR1

## Abstract

*Riemerella anatipestifer* mainly infects domestic ducks, geese, turkeys, and other birds, and causes considerable economic losses to the global duck industry. Previous studies have shown that concentrated cell-free culture filtrates of *R. anatipestifer* induce highly significant protection against homologous challenge. In this study, 12 immunogenic proteins were identified in the culture supernatant of *R. anatipestifer* strain Yb2 with immunoproteomic analysis. Of these, three immunogenic proteins, AS87_RS06600 (designated “PaR1” in this study), AS87_RS09020, and AS87_RS09965, which appeared in more than three spots on the western-blotted membrane, were expressed in *Escherichia coli* and purified. Animal experiments showed that the recombinant PaR1 (rPaR1) protein protected 41.67% of immunized ducklings against challenge with virulent Yb2, whereas rAS87_RS09020 or rAS87_RS09965 did not, and that ducklings immunized once with rPaR1 were 20, 40, and 0% protected from challenge with *R. anatipestifer* strains WJ4 (serotype 1), Yb2 (serotype 2), and HXb2 (serotype 10), respectively. In addition, rPaR1 immunized rabbit serum showed bactericidal activity against strain Yb2 at a titer of 1:8. These results indicate that rPaR1 of strain Yb2 protects against homologous challenge. Amino acid homology analysis show that PaR1 is a non-serotype-specific protein among different *R. anatipestifer* serotypes. Furthermore, PaR1 is mainly secreted outside the cell through the T9SS. Overall, our results demonstrate that *R. anatipestifer* PaR1 is a non-serotype-specific protective protein secreted by the T9SS.

## Introduction

*Riemerella anatipestifer*, the type species of the genus *Riemerella* in the family *Weeksellaceae* within the order *Flavobacteriales* in the phylum *Bacteroidetes* (Garcia-Lopez et al., 2019), is the causative agent of septicemia anserum exsudativa (Segers et al., 1993). It infects domestic ducks, geese, turkeys, and other birds. *R. anatipestifer* infection is probably the most economically important disease of farmed ducks worldwide. Once the bacterium infects a duck flock, it can become endemic and eradication can be difficult, with repeated infectious episodes possible. Currently, at least 21 serotypes of *R. anatipestifer* have been identified (Bisgaard, 1982; Pathanasophon et al., 2002; Cheng et al., 2003), but no or only little cross-protection has been observed among different serotypes of *R. anatipestifer* (Sandhu, 1979; Pathanasophon et al., 1996). Thus far, little is known about the protective antigens of *R. anatipestifer*.

Bacterial outer-membrane proteins are exposed on the cell surface, in direct contact with the host immune system, so some are potentially immunogenic antigens. The outer-membrane protein OmpA was the first immunogenic protein (Subramaniam et al., 2000) and virulence factor (Hu et al., 2011) identified in *R. anatipestifer*. The full length of the *ompA* gene is 1,467 bp, encoding 488 amino acids, and a 1,164 bp open reading frame (ORF) occurs within *ompA* at nucleotides (nt) 303–1,467. However, recombinant OmpA (residues 101–488) of serotype 15 *R. anatipestifer* strain 110/89 did not protect against challenge with this virulent serotype 15 strain, although specific antibodies were detected in the infected ducks (Huang et al., 2002). However, recombinant OmpA (residues 33–466) of serotype 2 *R. anatipestifer* strain Rf153 reportedly induced protection against challenge with both homologous and heterologous strains (60 and 50% protection against serotype 2 strain Rf153 and serotype 1 strain Rf63, respectively) (Zhai et al., 2013). In a recent study, we showed that the soluble recombinant protein rOmpA1164 (amino acids 102–488, encoded by a 1,164 bp ORF within *ompA*) but not rOmpA1467 (amino acids 1–488), provided partial protective immunity against homologous challenge (Xu et al., 2020). Outer-membrane protein GroEL is another immunogenic protein expressed by *R. anatipestifer*. Ducklings immunized with rGroEL were 50, 37.5, and 37.5% protected from the challenge with RA strains WJ4 (serotype 1), Th4 (serotype 2), and YXb-2 (serotype 10) (Han et al., 2012). In our previous study, an immunoproteomic analysis using duck antiserum against *R. anatipestifer* identified 34 immunogenic proteins, including OmpA and GroEL, among the whole-cell bacterial proteins of serotype 2 *R. anatipestifer* strain Th4, and the TonB-dependent outer-membrane receptor TbdR1 was shown to be a cross-immunogenic antigen among serotypes 1, 2, and 10 of *R. anatipestifer* (Hu et al., 2012). Further studies showed that TbdR1 is involved in hemin iron acquisition and necessary for optimal bacterial virulence (Lu et al., 2013). In another study, 12 immunoreactive proteins were identified in whole cells of *R. anatipestifer* serotype 2 strain RAf153 with an immunoproteomic analysis using rabbit antiserum against *R. anatipestifer*. One of these proteins, recombinant elongation factor G, responded to serum against strain RAf153, but not that from serotype 1 strain RjR1 (Zhai et al., 2012).

Formalin-inactivated *R. anatipestifer* bacterin has been shown to protect against challenge with virulent strains of homologous serotypes (Sandhu, 1979; Layton and Sandhu, 1984), whereas broth culture bacterin prepared from strains of serotypes 1, 6, 11, 14, and 19 conferred no significant protection against challenge with strains of heterologous serotypes. However, concentrated cell-free culture filtrates prepared from strain 1,081 of serotype 1 and strain 328 of serotype 19 induced highly significant protection against homologous challenge (Pathanasophon et al., 1996), indicating that the protective antigens exist on the outer membrane and in the culture supernatant of *R. anatipestifer*. In this study, the immunogenic proteins in the culture supernatant of *R. anatipestifer* strain Yb2 were identified with an immunoproteomic analysis, and PaR1, which is secreted by the T9SS, was identified as a novel protective antigen of *R. anatipestifer*.

## Materials and methods

### Bacterial strains, plasmids, and culture conditions

*Riemerella anatipestifer* strains WJ4 (serotype 1), Yb2 (serotype 2), and HXb2 (serotype 10) were isolated by Qinghai Hu from sick ducks in China in 2000 (Hu et al., 2010), and their full genome sequences have been deposited in GenBank under the accession number CP041029, CP007204, and CP011859. The suicide plasmid pYT354 (Zhu et al., 2017) was generously provided by Professor Mark J. McBride (University of Wisconsin–Milwaukee, Milwaukee, WI, USA). The *R. anatipestifer* strains and their derivatives were routinely grown in tryptic soybean broth (TSB; Difco, Detroit, MI, USA) at 37^°^C with shaking or on tryptic soybean agar (TSA). *Escherichia coli* cells were routinely grown in Luria–Bertani (*LB*) *broth* (Difco) at 37^°^C with shaking. The strains, plasmids, and primers used in this study are listed in Table 1. When required, antibiotics were added at the following concentrations: ampicillin (100 μg/mL), chloramphenicol (50 μg/mL), erythromycin (0.5 μg/mL), or kanamycin (50 μg/mL).

**TABLE 1:** Strains, plasmids, and primers used in this study.

Strains, plasmids or primers	Description^a^	References
**Strains**		
*R. anatipestifer* WJ4	The wild type strain, serotype 1, Kan^R^	(Hu et al., 2010)
*R. anatipestifer* Yb2	The wild type strain, serotype 2, Kan^R^	(Hu et al., 2010)
*R. anatipestifer* HXb2	The wild type strain, serotype 10, Kan^R^	(Hu et al., 2010)
*R. anatipestifer* Yb2ΔgldL	*gldL* deletion mutant of strain Yb2, T9SS defective	This study
*E. coli* S-17-1	Lpir hsdR pro thi; chromosomal integrated RP4-2 Tc:Mu Km:Tn7	Biomedal
**Plasmids**		
pYT354	Suicide plasmid, Ap^R^ (Em^R^)	(Zhu et al., 2017)
pYT354-gldL-LR	Recombinant suicide plasmid for deleting *gldL*	This study
**Primers for construction of suicide plasmid**		
gldL-L F	5′ TAGGATCCAGGATTGATAGGAGAATACCTTATTGA 3′ *Bam*HI site underlined	This study
gldL-L R	5′ CATGTCGACTACTACAGAAGCACCTACAGAGTAGAT 3′ *Sal*I site underlined	This study
gldL-R F	5′ AGTAGTCGACGCACTTTATGCATTACAGTTAGAA 3′ *Sal*I site underlined	This study
gldL-R R	5′ ATGCATGCTGTAAATCTAGTAGTTCCACATATCGT 3′ *Sph*I site underlined	This study
**Primers for identification of the** *gldL* **deleted mutant**		
16S rRNA F	5′ CAGCTTAACTGTAGAACTGC 3′	(Hu et al., 2011)
16S rRNA R	5′ TCGAGATTTGCATCACTTCG 3′	
ermF F	5′ GCCCGAAATGTTCAAGTTGT 3′	This study
ermF R	5′ TTTCCGAAATTGACCTGACC 3′	This study
gldL ORF F	5′ ATGAGACTAAATGATAAAACATTAAATT 3′	This study
gldL ORF R	5′ ATTTTATCCTTTCATCGCGTT 3′	This study

^a^Antibiotic resistance phenotypes are as follows: Kanamycin, Kan^R^; ampicillin, Ap^R^; erythromycin, Em^R^.

### Precipitation of cell-free culture supernatant of *R. anatipestifer* strain Yb2

The proteins in the culture supernatant of strain Yb2 were precipitated with the trichloroacetic acid (TCA)–acetone method, as described previously (Flaugnatti and Journet, 2017), with some modification. Briefly, *R. anatipestifer* strain Yb2 was grown to mid-log phase, at an optical density at a wavelength of 600 nm (OD_600_) of 1.0, in TSB at 37^°^C with shaking. The cells were pelleted by centrifugation at 7,000 × g for 10 min, and the culture supernatant was filtered through a 0.22 μm pore-size polyvinylidene difluoride (PVDF) filter (Merck Millipore, Darmstadt, Germany) to remove residual cells. TCA was then added to the filtered culture supernatant to a final concentration of 10% (w/v), mixed gently, and incubated at 4^°^C overnight. The precipitated proteins were collected by centrifugation at 16,000 × g for 1 h at 4^°^C, and the pellet was washed with cold acetone. The pellets were air dried and resuspended in solubilization buffer (7 M urea, 2 M thiourea, 4% CHAPS). The precipitated proteins were quantified with the Bradford method using Bio-Rad Protein Assay Dye Reagent (Bio-Rad, Hercules, CA, USA), and the samples were stored at -80^°^C until further analysis.

### Immunoproteomic analysis of proteins precipitated from culture supernatant

Samples containing 200 μg of proteins precipitated from culture supernatants were passively rehydrated onto 13 cm immobilized pH gradient IPG strips (GE Healthcare Life Sciences, Piscataway, NJ, USA) in a pH range of 3–10 for 12–16 h. The immunoproteomic analysis of the precipitated proteins was performed as described previously (Hu et al., 2012), and included isoelectric focusing (IEF), sodium dodecyl sulfate-polyacrylamide gel electrophoresis (SDS-PAGE), western blotting, image processing, matrix-assisted laser desorption/ionization time of flight mass spectrometry (MALDI-TOF MS), database searches, and bioinformatic analyses. Convalescent duck serum against *R. anatipestifer* strain Yb2 was used in the western blotting analysis.

### Expression and purification of selected proteins

In this study, proteins AS87_RS06600 (designated “PaR1”), AS87_RS09020, and AS87_RS09965 appeared in more than three spots on the western-blotted membrane in the immunoproteomic analysis. The genes encoding the three proteins were amplified from serotype 2 *R. anatipestifer* strain Yb2 and cloned into the pET28a vector (Novagen, Darmstadt, Germany). The recombinant proteins were expressed in *E. coli* BL21(DE3) cells (Novagen, Darmstadt, Germany) and purified with Ni-IDA affinity chromatography (DetaiBio, Nanjing, China) as described previously (Xu et al., 2020). The recombinant proteins rPaR1, rAS87_RS09020, and rAS87_RS09965 were detected on western blots probed with convalescent duck serum directed against *R. anatipestifer* strain Yb2.

### Evaluation of protective immunity induced by the recombinant proteins

One-day-old Cherry Valley ducklings were purchased from Lijia Duck Farm (Taixin, Jiangsu, China), and no serum antibodies against *R. anatipestifer* strains WJ4 (serotype 1), Yb2 (serotype 2), or HXb2 (serotype 10) were detected with a whole-cell ELISA (Prieto et al., 2003). The ducklings were housed in cages under a 12 h light/dark cycle with free access to food and water. All applicable international, national, and institutional guidelines for the care and use of the animals were followed. The protocol was approved by the Committee on the Ethics of Animal Experiments of Shanghai Veterinary Research Institute, Chinese Academy of Agricultural Sciences (permit number: SHVRI-ZD-2018-026).

Before immunization and challenge experiments, the median lethal doses (LD_50*s*_) for *R. anatipestifer* strains WJ4 (serotype 1), Yb2 (serotype 2), and HXb2 (serotype 10) were measured in ducklings as 1.97 × 10^8^ CFU, 1.07 × 10^6^CFU, and 1.89 × 10^5^ CFU, respectively. In this study, the immunized ducklings were challenged with 10 LD50 doses of each strain.

To evaluate the protective immunity conferred by the recombinant proteins rPaR1, rAS87_RS09020, and rAS87_RS09965, 8 days-old Cherry Valley ducklings were divided into four groups (12 ducklings per group) and immunized once intramuscularly with 100 μg of each recombinant protein or phosphate-buffered saline (PBS), each emulsified with Montanide ISA 70 V adjuvant (Seppic, Paris, France). At 2 weeks postimmunization, all the ducklings were challenged with 1 × 10^7^CFU of wild-type Yb2. Ducklings that became moribund were killed humanely and counted as dead, and then subjected to *R. anatipestifer* identification. The mortality of the ducklings was recorded daily for a period of 10 days after challenge.

To investigate whether rPaR1-immunized ducklings were protected from virulent challenge with *R. anatipestifer* strains of different serotypes, 8 days-old Cherry Valley ducklings were immunized with ISA-70-V-adjuvant-emulsified rPaR1 (*n* = 30 ducklings for three groups), ISA-70-V-adjuvant-emulsified PBS (*n* = 30 ducklings for three groups), or ISA-70-V-adjuvant-emulsified inactivated Yb2 bacterin (*n* = 10 ducklings). On day 15, the sera of the immunized ducklings from each group were collected and screened for rPaR1 antibodies with an ELISA, using 1 μg/mL rPaR1 as the coating antigen and *horseradish* peroxidase (HRP)-labeled rabbit anti-duck IgY (IgG) (Biodragon, Suzhou, China) as the secondary detection antibody. The rPaR1- and PBS-immunized ducklings (10 rPaR1-immunized and 10 PBS-immunized ducklings for each strain) were challenged with 2 × 10^9^ CFU of serotype 1 strain WJ4,10^7^ CFU of serotype 2 strain Yb2, or 2 × 10^6^ CFU of serotype 10 strain HXb2. Ten ducklings immunized with inactivated Yb2 bacterin were challenged with 10^7^ CFU of Yb2. The mortality of the ducklings was recorded daily, as above.

### Serum bactericidal assay

The natural ability of mouse complement (Sigma-Aldrich, St. Louis, MO, USA) and inactivated mouse complement (56^°^C for 30 min) to kill serotype 1 strain WJ4, serotype 2 strain Yb2, and serotype 10 strain HXb2, was determined. To each well of a 96-well flat-bottom plate (Corning, NY, USA) containing 45 μL of sterile PBS, 25 μL of mouse complement or inactivated mouse complement was added, and then 30 μL of each bacterial suspension (10^4^ CFU/mL in PBS). After incubation at 37^°^C for 30 min, 100 μL of the mixture was plated onto TSA plates. Percentage killing was calculated as the geometric mean value of [1-(CFU remaining after mouse complement/CFU remaining after inactivated complement)] × 100 from three trials, and the strain for which the natural bactericidal rate of mouse complement was <15% at a complement concentration of 25% (Martin et al., 2005) was used in the next bactericidal assay. Rabbit antiserum against rPaR1 and naïve rabbit serum were inactivated at 56^°^C for 30 min and 2-fold serial diluted in PBS. Then the bactericidal activity against serotype 2 strain Yb2 was performed as described previously (Gu et al., 1996). For each well of a 96-well flat-bottom plate, 50 μL of diluted rabbit antiserum against rPaR1 or naïve rabbit serum, 30 μL of bacterial suspension (10^4^ CFU/mL in PBS) and 15 μL of mouse complement serum were added. The plates were incubated at 37^°^C for 30 min, and 50 μL of the mixture in each well was spread onto TSA plates. The bactericidal assay was performed in triplicate. The highest serum dilution causing 50% killing was expressed as the bactericidal titer.

### Sequences analysis of *paR1* from *R. anatipestifer* strains of different serotypes

The nucleotide acid sequence of *paR1* (AS87_RS06600) from *R. anatipestifer* strains of different serotypes were retrieved from the GenBank database. The derived amino acid sequences of PaR1 from these strains were aligned and analyzed with Clustal W in the MegAlign program of the Lasergene 7.01 software (DNASTAR Inc., Madison, WI, USA).

### Preparation of rabbit antiserum against rPaR1

Rabbit antiserum against rPaR1 was obtained by immunizing 2 months-old New Zealand rabbits subcutaneously (s.c.) with 100 μg of purified rPaR1 protein emulsified with 50% (vol/vol) Montanide ISA 50 V adjuvant (Seppic) and boosted twice s.c. with 100 μg of rPaR1 at two weekly intervals. The rabbit sera were collected 7 days after the third immunization.

### Construction of type IX secretion system (T9SS)-defective mutant of *R. anatipestifer* strain Yb2

Most members of the phylum Bacteroidetes secrete proteins across the outer membrane *via* the type IX secretion system (T9SS) (Sato et al., 2010; McBride and Zhu, 2013). T9SS is a multiprotein system consisting of at least 20 proteins, and GldL is one of the core components of T9SS (Hennell James et al., 2021). To construct a T9SS-defective mutant, a 444 bp (nt 58–501) fragment of the strain Yb2 *gldL* ORF (687 bp) was deleted in-frame with the suicide plasmid pYT354, as described previously (Barbier et al., 2020). Briefly, a 2,599 bp region corresponding to the first 57 bp of *gldL* together with the directly upstream region was amplified with primers gldL-L P1 and gldL-L P2. A 2,773 bp region corresponding to the final 186 bp of *gldL* together with the directly downstream region was amplified with primers gldL-R P1 and gldL-R P2. The two fragments were ligated into suicide vector pYT354, generating the recombinant suicide plasmid p354-gldL-LR. This plasmid was then transferred into wild-type Yb2 by conjugation, and transformants were selected on TSA agar containing erythromycin and kanamycin. One positive colony was grown in TSB without antibiotics at 37^°^C overnight, and the cells were then plated on TSA containing 8% sucrose. The positive mutant colonies were screened by PCR using primers gldL ORF F plus gldL ORF R, and 16S rRNA F plus 16S rRNA R, to detect the 16S rRNA, and *gldL* ORF, respectively. A *gldL* deletion mutant, which was positive for 16S rRNA and has a smaller PCR product (about 250 bp) for amplification of *gldL* ORF than that in the wild type Yb2, was designated as Δ*gldL*.

### Subcellular localization analysis of PaR1

The subcellular localization of PaR1 predicted by PSORTb v.3.0^1^ was “unknown,” suggesting that *R. anatipestifer* may be not uniquely located.

To investigate whether the specific antibodies to rPaR1 in rabbit antiserum against rPaR1 could bound to the outer membrane of *R. anatipestifer* strainYb2, a whole-cell ELISA using whole Yb2 cells as the coating antigen was performed as described previously (Prieto et al., 2003). Naïve rabbit serum and convalescent duck serum against *R. anatipestifer* strain Yb2 were used as the negative and positive controls, and HRP-labeled goat anti-rabbit IgG (Solarbio, Beijing, China) and HRP-labeled rabbit anti-duck IgY (IgG) (Biodragon) as the secondary detection antibodies for detection of rabbit sera and duck serum, respectively. At the same time, PaR1-specific antibodies in the rPaR1 immunized rabbit serum and naïve rabbit serum were detected with an indirect ELISA using 1 μg/mL rPaR1 as the coating antigen. All samples were tested in triplicate. On the other hand, for indirect immunofluorescence assay, rabbit antiserum against rPaR1 was incubated with fresh whole Yb2 cells at room temperature for 2 h. After the cells were washed with PBS, the cells were incubated with Alexa Fluor 488-labeled goat anti-rabbit IgG antibody (ThermoFisher, USA) at room temperature for 1 h. After the washes, the cells were observed on a fluorescence microscope (Nikon Eclipse 80i, Japan). Rabbit serum against Yb2 and naïve rabbit serum were used as positive and negative controls.

The inner (cytoplasmic)-membrane proteins of wild-type Yb2 and the mutant Δ*gldL* were prepared as described previously (Spratt and Pardee, 1975), with some modification. Briefly, *R. anatipestifer* cells were grown to mid-log phase (OD_600_ = 1.0) in TSB at 37^°^C with shaking. The cells were centrifuged and washed twice with 0.01 M PBS (pH 7.0), and the pellet was resuspended in 0.01 M PBS (pH 7.0) containing 1 mM phenylmethanesulfonyl fluoride. After sonication, any unbroken cells were removed by centrifugation at 7,000 × g for 10 min. The membrane fraction was then collected by centrifugation at 100,000 × g for 30 min. The pellets were resuspended in 0.01 M PBS (pH 7.0) to which 1% (w/v) sarkosyl (Macklin, Shanghai, China) was added, and incubated at room temperature for 20 min. The membrane samples were then separated into the soluble (inner membrane) and insoluble (outer membrane) fractions by centrifugation at 100,000 × g for 30 min.

To determine whether protein PaR1 is secreted to the outside of *R. anatipestifer* cells through T9SS, the proteins in the culture supernatant of WT Yb2 and Δ*gldL* were precipitated with the TCA–acetone method, as described above. PaR1 was detected in the whole Yb2 cells, the inner membrane proteins, the normal cell-free culture supernatant, and the precipitated proteins in the culture supernatant with SDS-PAGE and western blotting, using rPaR1 immunized rabbit serum as the primary antibody. The blots were developed using an enhanced chemiluminescence detection kit (ECM Biotech, Suzhou, China).

### Statistical analysis

GraphPad Prism 9.0.0 software (GraphPad Software, Inc., San Diego, CA, USA) was used for statistical analysis and preparation of graphs. The statistical significance of the survival curves was determined with a log-rank (Mantel–Cox) test, and the significant differences of ELISA results between groups were identified using the two-tailed Student’s *t*-test. A probability (*p*) value of <0.05 was considered statistically significant.

## Results

### Immunoproteomics in the cell-free culture supernatant of *R. anatipestifer* strain Yb2

**FIGURE 1:**
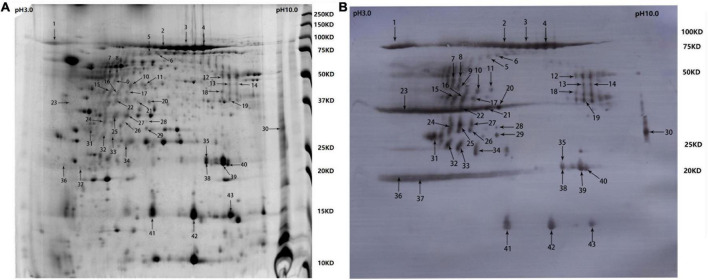
Two-dimensional electrophoresis (2-DE) gel and western blotting profile of cell-free culture supernatant of *R. anatipestifer* strain Yb2. **(A)** Separation of trichloroacetic acid (TCA)-precipitated proteins from the culture supernatant of *R. anatipestifer* Yb2 (200 μg) with 2-DE over pH 3–10 (stained with Coomassie Brilliant Blue G-250). Sites of excised spots are shown with arrows. **(B)** Western blotting analysis of the proteins on 2-DE gel, probed with convalescent duck serum against *R. anatipestifer* strain Yb2.

Samples containing 200 μg of proteins precipitated from the cell-free culture supernatant of *R. anatipestifer* strain Yb2 were separated with *two-dimensional* polyacrylamide gel electrophoresis (*2-DE)* and stained with Coomassie Brilliant Blue G-250 (Sigma-Adrich, MO, USA). The proteins were separated according to isoelectric point (PI) in the first dimension over a pH range of pH 3–10, and most proteins on the 2D gels had molecular weights (MWs) of 10–150 kDa (Figure 1A). The western blotting analysis detected 43 immunogenic protein spots when probed with convalescent duck serum against *R. anatipestifer* strain Yb2 (Figure 1B), but no appreciable spots appeared on the membrane probed with naïve duck serum (data not shown). Eleven immunogenic proteins were identified with MALDI-TOF-MS and a peptide mass fingerprinting (PMF) analysis (Table 2). Five proteins appeared in more than one spot, and of these, proteins AS87_RS06600 (PaR1), AS87_RS09020, and AS87_RS09965 appeared on 23, eight, and four spots, respectively. Moreover, both AS87_RS06600 and AS87_RS09020 were predicted to contain a type A carboxy-terminal domain (CTD) of the type IX secretion system (T9SS) (Kulkarni et al., 2017), suggesting they were secreted by T9SS. In addition, it is noteworthy that 11 immunogenic proteins identified in the culture supernatant of *R. anatipestifer* strain Yb2 in this study were not identified in the whole cells (Hu et al., 2012; Zhai et al., 2012) or culture supernatant (Zhai et al., 2013) of *R. anatipestifer* in immunoproteomic analyses in previous studies. The subcellular locations of these proteins predicted with the PSORTb v.3.0 software showed that six proteins (54.55%, 6/11) were extracellular proteins and the locations of five (45.45%, 5/11) were “unknown” (Table 2).

**TABLE 2:** Immunogenic proteins in the culture supernatant of *Riemerella anatipestifer* strain Yb2 identified by matrix-assisted laser desorption/ionization time of flight mass spectrometry (MALDI-TOF MS).

Spot no.	Protein name	GenBank accession no.	Locus tag on the Yb2 genome^a^	T9SS CTD^b^	Theoretical MW/PI	Protein score C. I. %	Subcellular location^c^
1–17, 19, 24–26, 30,32	Hypothetical protein (PaR1)	AKQ40033.1	AS87_RS06600	Type A CTD	117222.2/8.44	99.552–100	Unknown
31, 33, 36, 41–43	Carbohydrate-binding protein	AKQ40508.1	AS87_RS09020	Type A CTD	28565.6/9.19	100	Extracellular
35, 38–40	ABC transporter substrate-binding protein	AKQ40692.1	AS87_RS09965	–	25301.6/8.46	100	Extracellular
20–21	Hypothetical protein	AKQ40507.1	AS87_RS09015	–	44256.6/6.08	100	Extracellular
28–29	Hypothetical protein	AKQ40504.1	AS87_RS09000	–	34729.2/6.02	100	Unknown
11	Peptidase M16	AKQ38937.1	AS87_RS00925	–	49795.2/6.07	100	Extracellular
18	Hypothetical protein	AKQ40440.1	AS87_RS08675	–	48715.7/8.51	100	Extracellular
23	Hypothetical protein	AKQ40669.1	AS87_RS09845	–	104859.6/4.88	100	Extracellular
27	Hypothetical protein	AKQ39770.1	AS87_RS05240	–	34176.2/5.65	100	Unknown
34	Succinate-CoAligase	AKQ39333.1	AS87_RS02975	–	29662.3/5.34	100	Unknown
37	Alkyl hydroperoxide reductase	AKQ38855.1	AS87_RS00505	–	23820.8/4.94	100	Unknown

^a^The GenBank accession number of *R. anatipestifer* strain Yb2: NZ_CP007204.

^b^Most proteins that are secreted by the type IX secretion system (T9SS) have conserved carboxy terminal domains (CTDs) that belong to the protein domain family TIGR04183 (type A CTDs) or TIGR04131 (type B CTDs). The conserved domains of proteins were screened by CD-search program (https://www.ncbi.nlm.nih.gov/Structure/cdd/wrpsb.cgi).

^c^Subcellular locations were predicted by the PSORTb v.3.0 software (http://www.psort.org/).

### Expression and purification of PaR1, AS87_RS09020, and AS87_RS09965

In this study, three proteins (PaR1, AS87_RS09020, and AS87_RS09965), which appeared in more than three spots on PVDF membranes in a western blotting analysis, were expressed in *E. coli* and purified. The immunogenicity of the recombinant proteins was investigated with western blotting using duck antiserum against Yb2 as the primary antibody. The three recombinant proteins reacted with duck antiserum against Yb2 (Supplementary Figure 1), suggesting that they are immunogenic proteins of *R. anatipestifer*.

### Recombinant protein rPaR1 induced protective immunity against challenge

**FIGURE 2:**
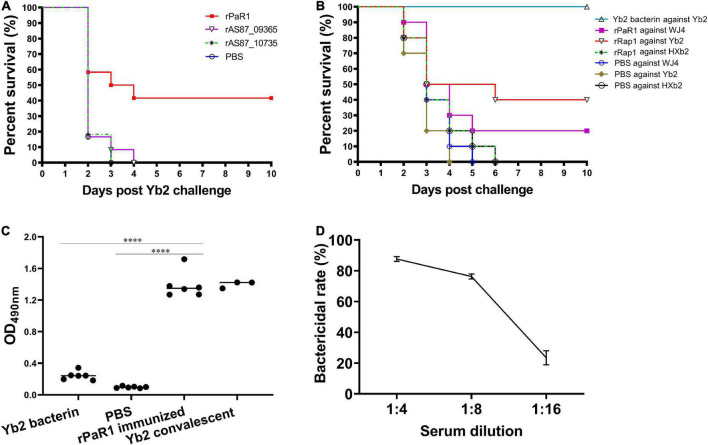
Recombinant protein recombinant PaR1 (rPaR1) confers protective immunity against Yb2 challenge. **(A)** Recombinant protein rPaR1, but not rAS87_RS09020 or rAS87_RS09965, conferred protective immunity against Yb2 challenge. **(B)** Recombinant protein rPaR1 conferred protective immunity against challenge with serotype 2 *R. anatipestifer* strain Yb2, but not serotype 1 strain WJ4 or serotype 10 strain HXb2. **(C)** ELISA scatter plots of antibodies to PaR1 detected in duck sera. Ducklings immunized with rPaR1, but not those immunized with inactivated Yb2 bacterin, displayed high levels of anti-PaR1 antibodies in their sera. Asterisks indicate statistically significant differences between two groups (^****^<0.0001). **(D)** Bactericidal activity of rabbit serum against rPaR1. Rabbit antiserum against rPaR1 and naïve rabbit serum were inactivated at 56^°^C for 30 min and 2-fold serial diluted in PBS. Then the bactericidal activity against serotype 2 strain Yb2 was performed. The highest serum dilution causing 50% killing was expressed as the bactericidal titer.

As shown in Figure 2A, five (41.67%) of 12 ducklings immunized once with the soluble rPaR1 protein were protected from wild-type Yb2 challenge, whereas all the ducklings immunized with rAS87_RS09020, rAS87_RS09965, or PBS died within 4 days of challenge. There was a significant difference in the protective immunity conferred by rPaR1 and the PBS control (*p* < 0.01). To investigate whether rPaR1 conferred protective immunity against challenge with *R. anatipestifer* strains of different serotypes, ducklings were challenged with serotype 1 strain WJ4, serotype 2 strain Yb2, or serotype 10 strain HXb2 at 2 weeks after rPaR1 immunization. All the ducklings inoculated with PBS emulsified with ISA 70 V adjuvant died within 6 days of challenge with strains WJ4, Yb2, or HXb2, whereas 2/10 (20%), 4/10 (40%), and 0% of ducklings immunized with rPaR1 emulsified with ISA 70 V adjuvant survived challenge with serotype 1 strain WJ4 (*p* > 0.05, versus PBS group challenge with WJ4), serotype 2 strain Yb2 (*p* < 0.05, versus group challenge with Yb2), and serotype 10 strain HXb2 challenge, respectively (Figure 2B), suggesting that rPaR1 did not confer protection against serotype 10 strain HXb2 or serotype 1 strain WJ4. Before the challenge, the sera from the immunized ducklings were collected and the PaR1-specific antibodies measured with an ELISA using rPaR1 as the coating antigen. As shown in Figure 2C, the average OD_490_ values for antibodies to PaR1 in the serum samples obtained from ducks immunized with ISA-70 V-emulsified rPaR1 was 1.39 ± 0.17, whereas that in sera from ducklings immunized with ISA-70 V-emulsified inactivated Yb2 bacterin was 0.24 ± 0.06.

The bactericidal activity of rPaR1 immunized rabbit serum was also determined. A natural bactericidal activity assay showed that the natural bactericidal rates of mouse complement against serotype 1 strain WJ4, serotype 2 strain Yb2, and serotype 10 strain HXb2 were below zero, 8.5% (<30%), and 87.4% (>30%), respectively. Therefore, the mouse complement used in this study was only suitable for the analysis of the bactericidal activity against serotype 2 strain Yb2, but not against serotype 1 strain WJ4 and serotype 10 strain HXb2. The bactericidal assay showed rPaR1 immunized rabbit serum showed bactericidal activity against strain Yb2 at a titer of 1:8 (Figure 2D).

### PaR1 is not a serotype-specific protein of *R. anatipestifer*

The *paR1* gene sequences of 33 *R. anatipestifer* strains of different serotypes were retrieved from GenBank. Among these, 26 strains carried one copy of the *paR1* gene, and the other seven strains (20190305E2-2, 20190109E1-1, 20190609E1-1, RCAD0416, 20190121E1-3, 20190507E1-1, 20190509E1-1) carried 2–3 copies of *paR1* homologous. A homology analysis showed that PaR1 of serotype 2 *R. anatipestifer* strain Yb2 shared 100% identity with those of strains RA-YM (serotype 1), RA-LZ01 (serotype 1), RA-GD (serotype 1), 17 (serotype 17), and RCAD0392, indicating that PaR1 is not a serotype-specific protein of *R. anatipestifer* (Supplementary Figure 2). In addition, PaR1 of strain Yb2 shared 99.9, 84.5, 83.8, and 85.5% identities with that of strains DSM15868 (= ATCC 11845, type strain), WJ4 (serotype 1), CH-2 (serotype 2), and HXb2 (serotype 10), respectively.

### PaR1 Protein localized to the culture supernatant of *R. anatipestifer*

**FIGURE 3:**
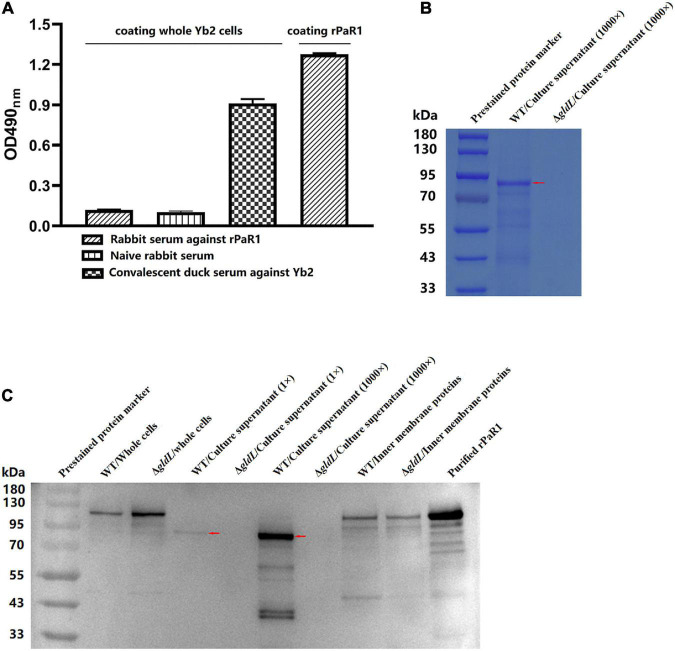
PaR1 protein localizes to the culture supernatant of *R. anatipestifer*. **(A)** Polyclonal antibodies against rPaR1 in immunized rabbit serum did not react with whole Yb2 cells in a whole-cell ELISA. **(B)** Protein secretion was defective in T9SS-defective mutant Δ*gldL*. *R. anatipestifer* cells were grown in TSB medium at 37^°^C with shaking and harvested in exponential phase (OD_600_ = 1.0). The cells were removed by centrifugation and filtered (0.22 μm). Equal volumes of cell-free culture supernatant of wild-type Yb2 (WT) and Δ*gldL* mutant were precipitated with TCA (concentrated 1,000-fold), and the proteins were separated with SDS-PAGE and stained with Coomassie Brilliant Blue G-250. The red arrow indicates the position of the main component in the trichloroacetic acid (TCA)-precipitated proteins from the culture supernatant of wild-type Yb2. **(C)** Mature PaR1 was detected with western blotting using rabbit antiserum against rPaR1 in the culture supernatant of wild-type Yb2, but not in that of T9SS-defective mutant Δ*gldL*. *R. anatipestifer* cells were grown in TSB medium at 37^°^C with shaking and harvested in exponential phase (OD_600_ = 1.0). Equal volumes of cultured WT and Δ*gldL* were used to prepared the whole cells, and inner membrane proteins. Proteins from equal volumes of cell-free culture supernatant of wild-type Yb2 (WT) and Δ*gldL* mutant were precipitated with TCA (concentrated 1,000-fold). The whole cells, normal cell-free culture supernatant, TCA-precipitated proteins from the culture supernatant (1,000-fold concentrated), and inner membrane proteins were analyzed for rPaR1 with SDS-PAGE and western blotting. The red arrows indicate the position of mature PaR1 in the TCA-precipitated proteins from the culture supernatant of wild-type Yb2.

To determine whether PaR1 is located on the outer membrane of *R. anatipestifer*, whole Yb2 cells were used as the coating antigen in a whole-cell ELISA. The average OD_490_ values for rabbit antiserum against rPaR1 was 0.088 ± 0.001, which was close to that for naïve rabbit serum, whereas that for rabbit antiserum directed against rPaR1 was 1.28 ± 0.02 when rPaR1 protein was used as the coating antigen in ELISA (Figure 3A), indicating that the polyclonal antibodies against rPaR1 could not bind to the whole *R. anatipestifer* cells. Furthermore, no fluorescence was observed on the *R. anatipestifer* cells under fluorescence microscopy when rabbit antiserum against rPaR1 was used as the primary antibody and fluorescein-isothiocyanate-labeled goat anti-rabbit IgG antibody as the secondary antibody (data not shown). These results indicate PaR1 does not localize to the *R. anatipestifer* outer membrane.

The proteins in the culture supernatant of Yb2 and T9SS-defective mutant Δ*gldL* were detected by SDS-PAGE and western blotting. Compared to the wild type, the amount of proteins in the cell-free culture supernatant of Δ*gldL* mutant was obviously reduced (Figure 3B), indicating that T9SS-defective mutant Δ*gldL* was defective in extracellular protein secretion. PaR1 of wild-type Yb2 (proPaR1) was predicted to be 117 kDa in size, according to its amino acid sequence. As shown in Figure 3C, a band close in size to proPaR1 and rPaR1 was detected with rabbit antiserum against rPaR1 from the whole cells of Yb2 and Δ*gldL* mutant with western blotting. In contrast, as shown in Figures 3B, C, and PaR1 was the major protein component in the culture supernatant of wild-type Yb2, and mature PaR1, which is about 85 kDa, was detected in the normal or 1,000-fold-concentrated cell-free culture supernatant of WT Yb2. At least six other smaller bands, including two obvious bands of about 40 kDa, were also detected. However, PaR1 was absent from both the normal and 1,000-fold-concentrated culture supernatant of the T9SS-defective mutant Δ*gldL*, indicating that PaR1 protein is mainly secreted to the outside of wild-type strain Yb2 cells *via* the T9SS. In addition, PaR1 was also detected among the inner-membrane proteins of Yb2 and mutant Δ*gldL*, and the most obvious bands had a molecular mass close to those of proPaR1 and rPaR1 (Figure 3C), suggesting the CTD is not cleaved yet for the inner membrane PaR1. The N-terminal signal peptide guides the PaR1 protein across the inner membrane in the first step in its secretion from *R. anatipestifer* cells, therefore, most probably, the PaR1 on the inner membrane is a normal intermediate state of the secretion process. Overall, the localization of PaR1 is extracellular after secretion through T9SS.

## Discussion

Identifying the immunogenic antigens is the first step in developing a novel generation of vaccines for *R. anatipestifer* infection and developing a method to detect the related antibody levels in duck sera. In general, bacterial outer-membrane proteins are considered potential immunogenic proteins and candidate protective antigens for vaccine development. However, in this study, our results showed that PaR1 is a protective antigen, although it is mainly secreted to the outside of *R. anatipestifer* by the T9SS.

The results in this study show that PaR1 of serotype 2 strain Yb2 shares 100% identity with that of other serotypes of *R. anatipestifer* strains, making cross-protection between different serotypes of *R. anatipestifer* strains possible. This is inconsistent with the long-held view that no or little cross-protection is afforded to *R. anatipestifer* strains of different serotypes (Sandhu, 1979; Pathanasophon et al., 1996). This may be attributable to the following factors: (1) The amino acid sequences of PaR1 may differ between two tested strains of different serotypes. Only when two PaR1 strains share the same protective antigenic epitope can there be cross-protection between two strains. (2) The localization of *R. anatipestifer* PaR1. PaR1 is mainly secreted outside the cell *via* T9SS, and the culture supernatant may be discarded during the preparation of inactivated *R. anatipestifer* bacterin. Therefore, no cross-protection induced by PaR1 was observed when ducklings were immunized with inactivated bacterin. For example, in the present study, the rPaR1-directed antibodies in the sera of ducklings immunized with Yb2 bacterin was very low. (3) The amount of PaR1 in the culture supernatant of *R. anatipestifer* was relatively low, so even when two *R. anatipestifer* strains shared 100% amino acid identity of PaR1, they did not induce enough cross-protection. Previous studies have shown that 10-fold-concentrated cell-free culture filtrates (10 × CF) of *R. anatipestifer* strains induced highly significant protection against homologous challenge (Pathanasophon et al., 1996). In the present study, high levels of antibodies to PaR1 were detected in the convalescent duck serum against *R. anatipestifer* strain Yb2 with an ELISA. Therefore, we speculate that if two different serotypes of attenuated *R. anatipestifer* vaccine strains, which have the same PaR1 amino acid sequence or the same protective antigenic epitope on PaR1, may provide cross-protection. Therefore, the amino acid sequence of PaR1 may be one of the factors to be considered when selecting attenuated candidate vaccine strains.

In this study, PaR1 was found to be the main immunogenic and protective protein in the cell-free culture supernatant, therefore, the effectiveness of concentrated culture supernatant in protecting against homologous challenge (Pathanasophon et al., 1996) may be attributable to PaR1, and thus this protection may be non-serotype-specific.

Proteins secreted by the T9SS have an N-terminal signal peptide that facilitates their export across the inner membrane by the Sec system and a conserved C-terminal domain (CTD), referred to as the CTD signal, that allows them to pass through the outer membrane *via* the T9SS (Kulkarni et al., 2017). In this study, the size of proPaR1 of strain Yb2 is about 117 kDa with an 18-amino-acid signal peptide at its N-terminus and a 78-amino-acid CTD (amino acids 962–1,039) at its C-terminus. Both the N-terminal signal peptide and the CTD are removed during the secretion of the protein into the culture supernatant *via* T9SS (Gorasia et al., 2015), and the remaining fragment is predicted to be 106 kDa, which is larger than the molecular mass of mature PaR1 in the culture supernatant (about 85 kDa). This suggests that the PaR1 protein in the culture supernatant had also been further proteolytically processed. Furthermore, at least six smaller and weaker bands were also detected with rabbit serum against rPaR1, suggesting that some of the mature PaR1 protein is further cleaved by protease(s). This explains why we detected PaR1 spots with different molecular mass in the immunoproteomic assay in this study. However, the protease that cleaves PaR1 and whether the smaller cleaved proteins have some other biological function remain unclear. This phenomenon is similar to that found in *Flavobacterium johnsoniae*, in which chitinase ChiA is also secreted by the T9SS. The predicted size of proChiA is 166 kDa, whereas that of secreted ChiA in spent medium is 92 kDa, and another 65 kDa band of ChiA was also detected (Kharade and McBride, 2014).

In summary, the results presented here demonstrate that *R. anatipestifer* PaR1 is a non-serotype-specific protective protein that is secreted outside *R. anatipestifer* cells *via* the T9SS.

## Data availability statement

The original contributions presented in this study are included in the article/Supplementary material, further inquiries can be directed to the corresponding author.

## Ethics statement

The animal study was reviewed and approved by the Committee on the Ethics of Animal Experiments of Shanghai Veterinary Research Institute, Chinese Academy of Agricultural Sciences (permit number: SHVRI-ZD-2018-026).

## Author contributions

QH designed the experiments and revised the manuscript. JW wrote the manuscript, performed serum bactericidal assay, and subcellular localization analysis of PaR1 and sequence analysis. YC performed immunoproteomic analysis, protein expression and purification, and construction of T9SS-defective mutant of strain Yb2. XH, JW, and YC did the animal experiments for evaluating protective immunity induced by the recombinant proteins. JW, XD, and YG analysis the data and drew the figures. XD, XS, and ZH participated in animal experiments and prepared the reagents and materials. All authors contributed to the article and approved the submitted version.

## Footnotes

1
https://www.psort.org/psortb/

## Supplementary material

Supplementary Figure 1 Confirmation of PaR1, AS87_RS09020, and AS87_RS09965 as immunogenic proteins of *R. anatipestifer*. The AS87_RS06600 (*paR1*), AS87_RS09020, and AS87_RS09965 ORFs of wild type Yb2 were cloned into the pET28a (+) vector and expressed in *E. coli* BL21(DE3) cells, respectively. The recombinant proteins were purified with Ni-IDA affinity chromatography. Then the purified recombinant proteins rPaR1, rAS87_RS09020, and rAS87_RS09965 were separated with SDS-PAGE and detected with western blotting using convalescent duck serum against *R. anatipestifer* strain Yb2.

Supplementary Figure 2 PaR1 is not a serotype-specific protein of *R. anatipestifer*. The *paR1* sequences of *R. anatipestifer* strains with different serotypes were retrieved from GenBank, and aligned and analyzed with Clustal W in the MegAlign program of the Lasergene 7.01 software. The locus tags of *paR1* in the genome of all bacterial strains, and their amino acid identities to PaR1 of *R. anatipestifer* strain Yb2, are listed.
