# Supplementary figures and images for: PaR1 secreted by the type IX secretion system is a protective antigen of Riemerella anatipestifer
Source: Front Microbiol. 2023 Jan 11;13:1082712. doi: 10.3389/fmicb.2022.1082712 (PMC9874225; doi:10.3389/fmicb.2022.1082712)

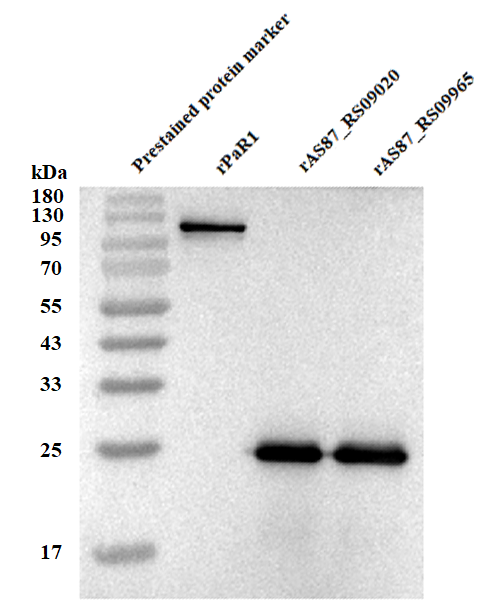

Supplement: Supplementary Figure 1 — Confirmation of PaR1, AS87_RS09020, and AS87_RS09965 as immunogenic proteins of R. anatipestifer. The AS87_RS06600 (paR1), AS87_RS09020, and AS87_RS09965 ORFs of wild type Yb2 were cloned into the pET28a (+) vector and expressed in E. coli BL21(DE3) cells, respectively. The recombinant proteins were purified with Ni-IDA affinity chromatography. Then the purified recombinant proteins rPaR1, rAS87_RS09020, and rAS87_RS09965 were separated with SDS-PAGE and detected with western blotting using convalescent duck serum against R. anatipestifer strain Yb2. [file Image_1.TIF]

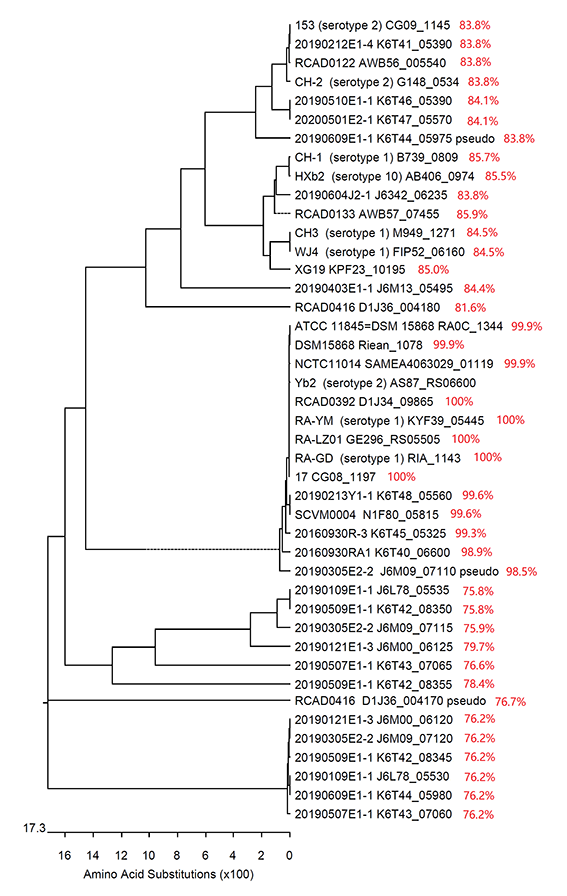

Supplement: Supplementary Figure 2 — PaR1 is not a serotype-specific protein of R. anatipestifer. The paR1 sequences of R. anatipestifer strains with different serotypes were retrieved from GenBank, and aligned and analyzed with Clustal W in the MegAlign program of the Lasergene 7.01 software. The locus tags of paR1 in the genome of all bacterial strains, and their amino acid identities to PaR1 of R. anatipestifer strain Yb2, are listed. [file Image_2.TIF]
